# Analysis of within Subjects Variability in Mouse Ultrasonic Vocalization: Pups Exhibit Inconsistent, State-Like Patterns of Call Production

**DOI:** 10.3389/fnbeh.2016.00182

**Published:** 2016-09-28

**Authors:** Michael A. Rieger, Joseph D. Dougherty

**Affiliations:** ^1^Department of Genetics, Washington University School of MedicineSt. Louis, MO, USA; ^2^Department of Psychiatry, Washington University School of MedicineSt. Louis, MO, USA

**Keywords:** mouse ultrasonic vocalization, mouseTube, state vs. trait, linear mixed models, mouse behavior, mouse development

## Abstract

Mice produce ultrasonic vocalizations (USV) in multiple communicative contexts, including adult social interaction (e.g., male to female courtship), as well as pup calls when separated from the dam. Assessment of pup USV has been widely applied in models of social and communicative disorders, dozens of which have shown alterations to this conserved behavior. However, features such as call production rate can vary substantially even within experimental groups and it is unclear to what extent aspects of USV represent stable trait-like influences or are vulnerable to an animal's state. To address this question, we have employed a mixed modeling approach to describe consistency in USV features across time, leveraging multiple large cohorts recorded from two strains, and across ages/times. We find that most features of pup USV show consistent patterns within a recording session, but inconsistent patterns across postnatal development. This supports the conclusion that pup USV is most strongly influenced by “state”-like variables. In contrast, adult USV call rate and call duration show higher consistency across sessions and may reflect a stable “trait.” However, spectral features of adult song such as the presence of pitch jumps do not show this level of consistency, suggesting that pitch modulation is more susceptible to factors affecting the animal's state at the time of recording. Overall, the utility of this work is three-fold. First, as variability necessarily affects the sensitivity of the assay to detect experimental perturbation, we hope the information provided here will be used to help researchers plan sufficiently powered experiments, as well as prioritize specific ages to study USV behavior and to decide which features to consider most strongly in analysis. Second, via the mouseTube platform, we have provided these hundreds of recordings and associated data to serve as a shared resource for other researchers interested in either benchmark data for these strains or in developing algorithms for studying features of mouse song. Finally, we hope that this work informs both interpretation of USV studies in models of developmental disorder, and helps to further research into understanding the neural processes that contribute to the production and predictability of USV behavior.

## 1. Introduction

The ultrasonic vocalizations (USV) of young mouse pups in response to maternal isolation has been studied for over five decades (Sewell, 1970; Smith, 1976; Ehret, 1980; Elwood and Keeling, 1982; Hahn et al., 1998; Hofer et al., 2002). The ability of isolation to elicit pup USV begins within days of birth and shows a peak in early postnatal development followed by a steady decline until 2 weeks of age (Hahn et al., 1998). These vocalizations function as a simple form of communication as they stimulate search and retrieval behavior from dams (Smith, 1976; D'Amato and Populin, 1987; Hahn and Lavooy, 2005). Because pup USV is easily elicited in the laboratory (Hofer et al., 2002), and amenable to automated analysis (Holy and Guo, 2005; Burkett et al., 2015), it has been assessed routinely as an anxiety- and communication-related phenotype in models of neurodevelopmental disorder (Branchi et al., 2001; Scattoni et al., 2009). A number of knockout mouse lines for autism spectrum disorder (Scattoni et al., 2008b; Dougherty et al., 2013; Yang et al., 2015), as well as for speech and language disorder risk genes (Fujita et al., 2008) and stuttering (Barnes et al., 2016), show changes to pup USV. These include either changes in the rate of USV production, or other spectral or temporal features of vocalization. Although this behavior is not human language, pup USV is a robust milestone of early postnatal development, and isolation-induced infant vocalization is a conserved behavior across mammals (Elliot and Scott, 1961; Ehret, 1980; Motomura et al., 2002; Shair, 2007; Stoeger-Horwath et al., 2007). Thus, understanding the neurobiological mechanisms mediating deficits of pup USV in disease models may help elucidate some conserved biology underlying these disorders of neurodevelopment.

Though production of USV is typically a robust behavior across a litter of animals, individual mouse pups show substantial variability, ranging from 0 to several hundred calls in a typical recording of wildtype C57BL/6J animals during the first week of life. Although most studies of USV in neurodevelopmental disorder models focus on mean differences between experimental and control groups, it is not often reported how variable this behavior is between and within subjects. While two mice of an inbred line are assumed to possess identical genetic backgrounds, this does not preclude a large degree of individual difference in behavioral expression (Chesler et al., 2002; McClearn, 2006; Ramos, 2008). The relative degree of inter- and intra-individual variation provides an estimate of the consistency or predictability of USV. The utility of estimation of the consistency of behavior and modeling intra-individual variation has been recognized in human clinical studies (Vangeneugden et al., 2004), human psychology (Mroczek and Spiro, 2003; Hoffman, 2007), and ecology, but such variability is not typically reported in studies of mouse USV, though it has been explored in the vocalizations of other species (Boncoraglio and Saino, 2008; Roulin et al., 2009). In human personality theory, it has been useful to consider the differences between “trait” vs. “state” influences on behavior: a state is a transient condition that influences behavior (e.g., feeling fear when seeing a snake), while a “trait” is a more stable aspect of personality that has a durable influence on behavior across time and situations (e.g., being a generally anxious person) (Dall et al., 2004; Spielberger, 2010). Borrowing these terms, individual-level behavioral expression patterns in USV might be due to any number of uncontrolled covariates that could mediate either state-like or trait-like differences in behavior. These include differences in intra-uterine environments and maternal health during pregnancy (Venerosi et al., 2009; Malkova et al., 2012; Golub et al., 2016) or maternal experience and quality of care (feeding, licking, etc.) (Thornton et al., 2005), which might have stable, trait-like impacts. Additionally, extrinsic factors such as degree of handling during the assay and temperature of the assay chamber (Hofer et al., 2002), maternal behavior just prior to the assay, or physiological variables (hunger/satiety, heart rate, breathing, etc.) may have a more immediate impact. Only a subset of these external factors can be reasonably measured during the course of an experiment. For example, typical USV protocols call for controlling temperature using an incubator or a heating pad before recording, as well as minimizing handling (Hofer et al., 2002). However, even if all such factors could be controlled, some aspects of USV may yet exhibit stochasticity. Such “randomness” in behavior is demonstrable even in simpler organisms. In *C. elegans*, although the average response of worms is to move toward an attractive olfactory stimulus, individual worms deviate from the expected pattern. In this organism, this has been shown to be controlled by neural states, where specific neurons control apparent randomization of the output behavior (Gordus et al., 2015). In mice, integration of enviromental covariates and intrinsic neuronal states may differ between time points and individuals, generating a variable amount of produced USV.

Furthermore, USV is a data-rich behavioral response with numerous features in the spectral and temporal domains of audio. In particular, some features of USV may be highly consistent within an animal relative to the population across days, showing a strong “trait”-like influence on variability. Other features may be more consistent within a recording session, but display high levels of intra-individual variability across days, perhaps reflecting an individual mouse's acute “state” on a given day. Finally some features may yet remain unpredictable even within a recording session. These degrees of consistency within and between individuals may reflect differential susceptibilities among features of USV to genetic, environmental, and intrinsic neuronal factors, leading some behaviors to show more stable “trait”-like influences (high consistency across days) while others might show patterns of variation more consistent with “state”-like responses (low consistency across days). Importantly, prior studies of features of pup USV have not considered the consistency of individuals, and determining whether a feature is more state- or trait-like may alter both interpretation of findings in disease models and the search for neurobiological mediators of pup USV.

Thus, to address the concept of consistency in USV behavior, we have used mixed modeling statistical approaches. Linear mixed models (LMMs) have proven a powerful way to estimate behavioral consistency patterns by partitioning random variance terms which describe the degree of inter- and intra-individual variability. In this study, we have employed the mixed model intra-class correlation (ICC) coefficient (Vangeneugden et al., 2004, also referred to as “repeatability” Nakagawa and Schielzeth, 2010, 2013) in order to understand consistency in features of USV across three independent discovery cohorts, totaling 285 subjects, and across two strains: FVB/AntJ and C57BL/6J (“Pooled Cohort Study,” PCS). We analyzed call rate (calls per minute), spectral, and temporal features of USV across three time points during postnatal development after controlling for effects of animal strain, age, and relative size. We also analyzed these features binned within recording session at each postnatal time point in order to understand consistency within a session. In order to validate our findings, we recorded additional litters of each strain at high temporal density (postnatal days 3–14, “Time Course Study,” TCS) as a replication study and to further probe the temporal dynamics of consistency. We found that despite clear group-level changes (due to age or strain) in both discovery and replication cohorts, features nevertheless varied in consistency across development, with some features, such as call rate, being largely unpredictable from day to day for a given animal. Within session however, we found that most features of USV exhibited significantly higher consistency on any given postnatal day. Furthermore, some features that showed low consistency over postnatal days, such as USV call rate, demonstrated a narrow window of high consistency near the peak of USV behavior. Early postnatal development is a highly dynamic time period for pups. To explore whether features of USV exhibit more stable behavior across measurements after animals have fully developed, we additionally looked at consistency in features of USV exhibited during adult male-female encounters. In contrast to pup USV, some features of adult USV showed dramatically higher consistency across test days, including the rate of ultrasonic calling and average call duration. Remaining features, such as the fraction of calls containing instantaneous jumps in pitch, did not show increased consistency. Thus, while the amount of USV produced by an animal may acquire trait-like stability later in life, other features remain dependent on the state of the animal, environmental context, or other influences.

## 2. Methods

### 2.1. Animals

All protocols involving animals were approved by the Animal Studies Committee of Washington University in St. Louis. Animals for pooled cohort study (PCS) consisted of 133 C57BL/6J in Cohort 1 (18 litters of median size 8 animals, ranging from 4 to 11 animals per litter), 105 C57BL/6J in Cohort 2 (15 litters of median size 8 animals, ranging from 2 to 9 animals per litter), and 47 FVB/AntJ (Jackson Laboratory strain FVB.129P2-Pde6b(+)Tyr(c-ch)/AntJ, 004828) in Cohort 3 (5 litters of median size 10, ranging from 6 to 12 animals). Animals in Cohorts 1 and 2 were originally planned to determine the effect of conditional knockout of the Celf6 gene in dopaminergic or GABA-ergic neurons on USV, and were generated by crossing Celf6^*flox*/*flox*^ X Celf6^*flox*/*wt*^; DAT-Cre (Jackson Laboratory strain B6.SJL-Slc6a3^*tm*1.1(*cre*)^Bkmn/J) or Celf6^*flox*/*flox*^ X Celf6^*flox*/*wt*^; VGAT-Cre (Jackson Laboratory strain Slc32a1^*tm*2(*cre*)*Lowl*^/J). No Celf6 genotype effects were detected on any USV metric scored (See Supplemental Figures 1, 2), and these data were pooled across genotype for the present analysis. Nonetheless, for the follow-up time course study (TCS) looking at vocalization every day postnatally between days 3 and 14, we used 13 wild-type C57BL/6J and 13 FVB/AntJ (Jackson Laboratory) from two litters each, of 8 and 5 respectively. Animals were maintained in a barrier facility. Breeding cages consisted of a single male and a single female, and both parents were present during pregnancy, birth, and during the time of assay. Cages were maintained by our facility on a 12 : 12 hr light:dark schedule with food and water supplied *ad libidum*. Adult mice were composed of 47 C57BL/6J males and 41 females aged 7–11 weeks. Adult mice were originally planned to determine the effect of global knockout of the Celf6 gene on adult USV in male-female dyadic interactions. No Celf6 genotype effects were detected on any USV metric scored (Supplemental Figure 3), and data were pooled across genotype for the present analysis.

### 2.2. USV recording and processing

#### USV recording—pups

Ultrasonic vocalization for Cohorts 1, 2, and 3 (PCS) was recorded on postnatal days 5, 7, and 9. For follow-up study (TCS), recordings were performed every day postnatally from days 3 through 14. All recordings were performed in the afternoon between 12:00 and 17:00. On first day of recording, subjects were each marked for identification immediately after recording by toe clip (PCS) or tattooing (TCS, Aramis Micro Tattoo Kit, Ketchum). On following days, subjects were recorded in random order and identifying marks were noted after recording, along with sex and weight. At the time of recording, a litter is separated from its parents by placing the parents in a temporary cage. The entire home cage with litter undisturbed is placed in an incubator and allowed to rest for 10 min. The pups' external temperature is regularly monitored with an infrared temperature gun digital thermometer (HDE-B01, HDE) and the incubator is maintained such that external temperature remains between 31 and 34°C. If the external temperature deviates below 30°C, the incubator is adjusted until external temperature returns within range, in order to minimize effects of cooling the pups on USV. For recording a pup, the pup is moved with minimal handling into an anechoic, sound attenuating chamber (Med Associates Inc.) and audio is recorded for 3 min using a CM16 microphone (Avisoft Bioacoustics), amplified and digitized using UltraSoundGate USG116H, using a gain of 1.4 dB, 250 kHz sampling rate, bit depth of 16, using Avisoft RECORDER software.

#### 2.2.1. USV recording—adult M-F dyads

Adult male animals were generated from group-housed weaned juveniles and were singly housed 24 h before test time. Females were maintained group-housed, between 4 and 5 animals per cage. The testing chamber consisted of an empty mouse cage (no bedding) placed inside an anechoic, sound attenuating chamber (the same used for pup testing). Testing occurred during the beginning of the animals' dark cycle (between 18:00 and 20:00), and proceeded as follows: (1) Habituation phase: males were placed in the test environment for 10 min with concurrent recording of USV as in the case of pup recordings. No USVs were detected for males during the habituation phase. (2) Test phase: A stranger female was added to the test environment and the dyad was recorded for 10 min. After testing, males were returned to single housing, and the test environment was cleaned with 70% ethanol followed by 2% Nolvasan solution (Zoetis Inc.) in between each animal. The number of days between tests was allowed to vary between 1 and 7 days, and the median number of intervening days was 4. No significant effect of the number of intervening days between test days on USV features was detected. Each male was tested on 2 days, with a different female each day. Pup and adult audio files were processed using the same computational pipeline.

#### 2.2.2. White noise filtering in the frequency domain

An automated method was designed to filter noise and improve automated call detection. A 10-s chunk is chosen at random from each audio file. The fast Fourier transform (FFT) is performed using 512 FFT bins corresponding to $\frac{512}{2}+1\;=\;257$ audio frequencies ranging from 0 to 125 kHz, and 50% temporal overlap corresponding to a temporal resolution of $0.5\;\cdot \;\frac{250000}{512}=1.024x10^{-3}$ s. A histogram of log_10_(FFT magnitude) is computed for all magnitudes in FFT bins corresponding to frequencies between 20 and 120 kHz. The main bulk of this histogram corresponds to the noise level in the spectrum which is assumed to be Gaussian in distribution. The mean of the noise distribution is estimated to be the peak of this histogram and a threshold is set at μ_*noise*_+2.5σ where only spectral magnitudes greater than threshold are designated as signal. This reliably separates the baseline of the FFT magnitudes from signal peaks for pup and adult calls. Such a threshold is determined for each file individually, however thresholds varied little across all files indicating a relatively constant background recording environment [not shown]. The noise distribution was estimated between 20 and 120 kHz since all sound outside of this range is band-pass filtered.

#### 2.2.3. Spectrogram preparation and band-pass filtering and automated call detection

Spectrogram preparation and automated call detection were performed in MATLAB using code adapted from Holy and Guo (2005). Briefly, after determining a threshold for white noise, the entire FFT (512 bins, 50% overlap, time resolution 1.024 ms, frequency resolution 488.2 Hz) is computed for each file, where magnitude < threshold is set to 0 and sound is band-passed filtered to reside within 20–120 kHz. All sound < 20 kHz and >120 kHz is also set to zero. Ultrasound calls are detected using thresholds of 5 ms minimum duration, 0.15 minimum spectral purity, 1.0 maximum spectral discontinuity, with gaps < 30 ms between adjacent calls merged. In Holy and Guo (2005), 0.25 spectral purity was suggested as appropriate threshold. Empirically we have determined that 0.15 is more reliable and results in fewer instances where spectrally impure parts of longer calls lead to a call artificially scored as two calls. After automated call detection, random subsets of spectrograms (10–20% of all files) are inspected manually to ensure that automated scores overlap with human-distinguishable calls observed in the spectrogram.

#### 2.2.4. Call feature extraction

After calls are detected, features for each call are extracted as follows. The dominant frequency (“pitch”) is determined for each 1.024 ms time bin in the spectrogram for each call by determining the FFT bin with maximum power (Power ∝ magnitude^2^). The median pitch is determined, as well as the total duration of each call. The presence of discontinuous jumps in pitch was determined as changes over time greater than ±10 kHz. Calls can also contain harmonic frequencies; these were not analyzed. The inverse FFT was computed from each call's spectrogram to yield the noise- and frequency-filtered waveform. A smoothed waveform envelope was estimated by computing a windowed RMS amplitude (512 samples, 50% overlap). The peak RMS amplitude was extracted from this envelope and power was computed as dB ref 1.0. The CM16 microphone was not calibrated, thus dB SPL were not computed, but dB are expressed with full-scale reference [max = 0 dB, dB = $10\cdot \log_{10}\left({\text{full scale amplitude}}^{2}\right)$].

### 2.3. Statistical analysis

Univariate LMMs for each feature of USV were computed using the *lme4* package (Bates et al., 2014) in R (Team, 2013) fitting a random intercept model grouped by subject id. Models were fitted using strain, postnatal day, and animal size as fixed effect factors. Postnatal day and animal size both entered models as continuous variables. Postnatal day was recentered at day 7 and fitted for both linear and quadratic effects in order to account for the “inverted U” pattern in development with a rise, peak, and fall in behavior. Animal size was z-score normalized weight with respect to day and strain as raw weight itself varies with both. For adults, only test day was used as a fixed effect factor. Significance of main effects and interactions in the data were computed by likelihood ratio tests (analysis of deviance) on nested models of increasing complexity using the anova() function in R.

Call rate (calls per minute) was transformed as the natural logarithm before modeling ($\log\frac{calls+1}{minutes}$). Other USV features were not transformed. We also fitted call rate using a negative binomial generalized linear mixed model (NB-GLMM). Fixed effect coefficients between the LMM on log-transformed call rate and the NB-GLMM on untransformed count data were highly similar (Pearson's *R* = 0.99). The log-transformed model was used in order to compare mixed model parameters across all features of USV fitted with the same algorithm. The ICC coefficient was determined using the fitted point estimates of random intercept variance ($\sigma_{\alpha }^{2}$) and residual error variance ($\sigma_{\varepsilon }^{2}$) from the the LMMs as described in Results.

In order to determine confidence bounds for model parameters, we employed a parametric bootstrap procedure. Using the point estimates of $\sigma_{\alpha }^{2}$ and $\sigma_{\varepsilon }^{2}$ as starting points, the *i*th bootstrap sample y*^(*i*)^were computed as:

$$\begin{array}{l} y{*}^{\left(i\right)}=X^{\left(i\right)}\cdot \beta +rnorm\left(mean=0,sd=\sqrt{\sigma_{\alpha }^{2}}\right)\\ \;+\;rnorm\left(mean=0,sd=\sqrt{\sigma_{\varepsilon }^{2}}\right) \end{array}$$

where *X*^(*i*)^ is the *i*th row of the fixed effects design matrix and β is the vector of fixed effects coefficients. Thus, *X*^(*i*)^β represents the expected value E(y) for the *i*th observation, which is then perturbed by drawing a random intercept and error from normal distributions [the R rnorm() function] with means of 0 and standard deviation as the square-root of the fitted LMM variance estimates. Each vector y^*^ represents a bootstrap sample dataset. The LMM was re-fitted using each y^*^ sample dataset for 100,000 iterations. The 95% confidence bounds for fixed effect coefficients, $\sigma_{\alpha }^{2}$, $\sigma_{\varepsilon }^{2}$, and the ICC were determined as the lower 2.5% and upper 97.5% quantiles of the bootstrap distribution. This procedure is preferable to a strict resampling with replacement of the original values of y, as it does not result in bootstrap sample datasets lacking factor levels and leaves the fixed effect correlation structure intact.

To compute values of ICC within session, recordings were binned into 3 × 1-min bins and USV aggregate features (e.g., average duration) were recomputed for each bin. LMMs were fitted on each postnatal day using strain and bin number as categorical variables, and z-score normalized weight as previously. ICC values obtained from within session calculations were compared to ICC values compared from calculations across postnatal days using a non-parametric Wilcoxon rank-sum test.

In order to explore consistency graphically across all USV features (regardless of scale, **Figures 3, 4, 6**), we computed Studentized residuals. Residuals from the full model take into account both fixed and random effects, and as such are not useful for looking directly at consistency as any consistent patterns expressed in the random intercepts have been removed. Thus, we computed a first-level residual where a residual $\tilde{\varepsilon }$ is the result of a data point *y*^(*i*)^ adjusting for the model's expected value *E*(*y*)^(*i*)^ (not taking into account random effects) as:

$${\tilde{\varepsilon }}^{\left(i\right)}=y^{\left(i\right)}-E{\left(y\right)}^{\left(i\right)}$$

**Figure 1:**
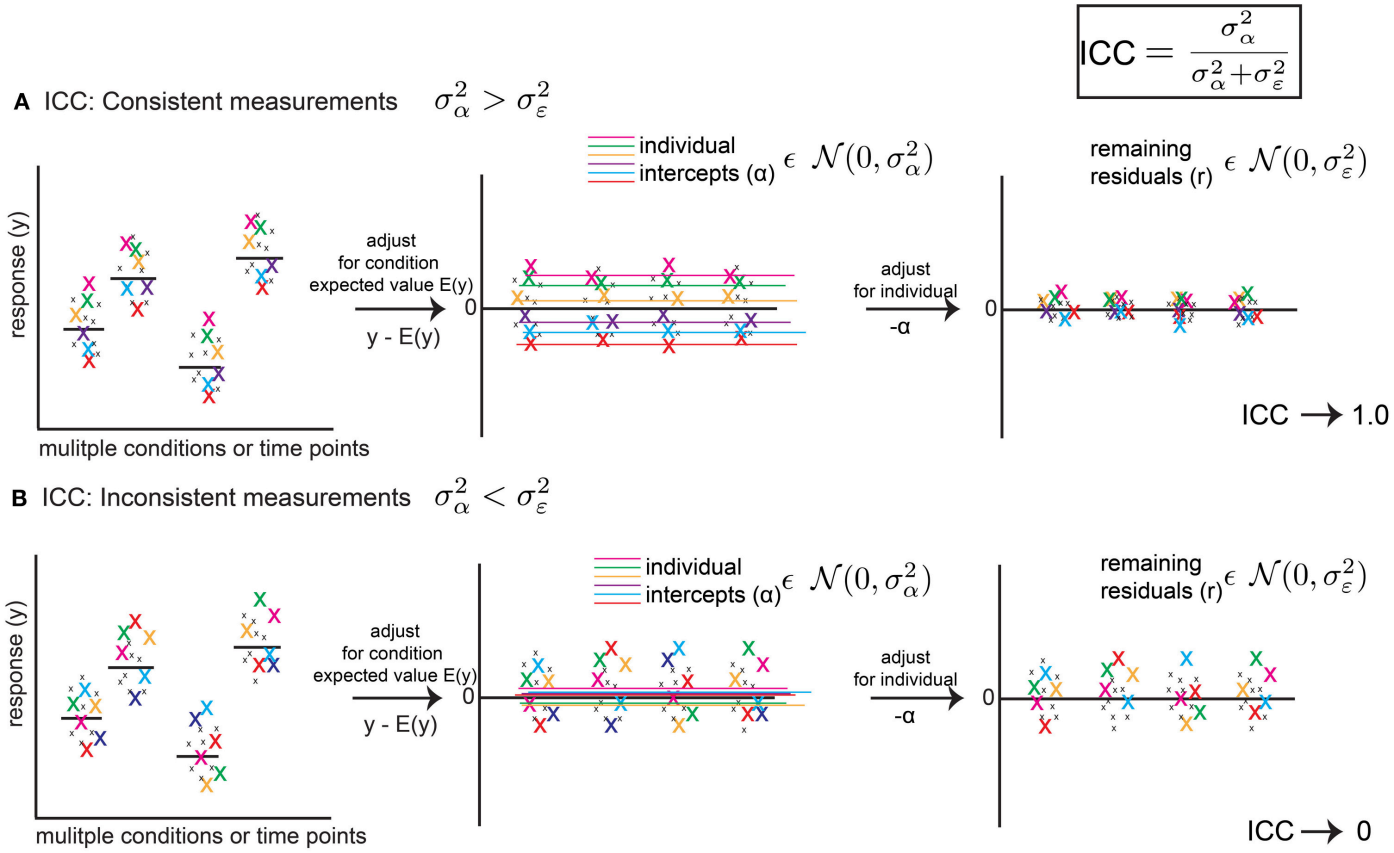
**The Intra-Class Correlation (ICC) defined from a linear mixed model (LMM) reflects the level of behavioral consistency of individual animals across multiple measurements**. The ICC (upper right) is defined as $\sigma_{\alpha }^{2}/\left(\sigma_{\alpha }^{2}+\sigma_{\varepsilon }^{2}\right)$, using mixed model random variance terms, and represents consistency of behavior across multiple measurements, where $\sigma_{\alpha }^{2}$ is the random effect variance term and $\sigma_{\varepsilon }^{2}$ is the error variance term from the LMM (fitting a random intercept only, as a function of animal identity). **(A)** Hypothetical scenario showing how the ICC reflects a consistent pattern. Left Panel: a response variable (e.g., rate of ultrasonic calls per minute) is measured for the same six animals(color coded x's) across conditions and time points. Middle Panel: After adjusting for expected values E(y) (e.g., group means or regression predictions for variables such as age or strain), random intercepts reflect an average expectation for a particular animal's position in the distribution of residuals. If measurements are consistent, the variance of these intercepts ($\sigma_{\alpha }^{2}$) reflects most of the remaining variance in the data. Right Panel: After adjusting for intercepts α, residuals are squeezed toward zero. Thus, $\sigma_{\alpha }^{2}\gg \sigma_{\varepsilon }^{2}$ and the ICC approaches 1.0. **(B)** Hypothetical scenario showing inconsistent measurements. After adjusting for time point or condition (Left Panel), residuals (Middle Panel) vary inconsistently from measurement to measurement for a given animal, and average values across measurements (random intercepts) are close to zero, and $\sigma_{\alpha }^{2}$ is small and reflects little of the remaining variance in the data. After adjusting for random intercepts, the residuals are mostly unchanged (Right Panel). Thus, $\sigma_{\varepsilon }^{2}\gg \sigma_{\alpha }^{2}$ and the ICC approaches 0. Thus, the ICC is a metric which summarizes consistency of patterns of behavior across measurements. The ICC is a point estimate, but using a bootstrap procedure we are able to assign confidence intervals to the ICC.

Such a residual is represented in the middle panel of Figures 1A,B and has units that are the same as the units of *y*. To normalize for units, a Studentized residual was computed as:

$$z^{\left(i\right)}=\frac{{\tilde{\varepsilon }}^{\left(i\right)}}{\tilde{\sigma }\left(1-h^{\left(i\right)}\right)}=\frac{y^{\left(i\right)}-E{\left(y\right)}^{\left(i\right)}}{\tilde{\sigma }\left(1-h^{\left(i\right)}\right)}$$

where $\tilde{\sigma }\left(1-h^{\left(i\right)}\right)$ is the estimate of the standard deviation at ${\tilde{\varepsilon }}^{\left(i\right)}$. We took $\tilde{\sigma }$ as the estimate of the model standard error before partitioning variance:

$$\tilde{\sigma }=\sqrt{\sigma_{\alpha }^{2}+\sigma_{\varepsilon }^{2}}$$

and *h*^(*i*)^ is the *i*th diagonal entry from the hat matrix **H**:

$$\text{H}=X{\left(X^{T}X\right)}^{-1}X^{T}$$

and *h* = *diag*(**H**). Thus, *z*^(*i*)^ represents the linear modeling analog to a z-score (e.g., $\frac{y^{\left(i\right)}-\bar{x}}{sd^{\left(i\right)}}$) and has units of standard deviation.

## 3. Results

### 3.1. Assessment of consistency of USV features across early postnatal development

In order to examine consistency, we have employed the ICC coefficient defined from the LMM. For a LMM of a response *y* (e.g., a feature of USV such as call duration), modeling fixed effects and a random intercept, we have a model of the form:

$$y^{\left(i\right)}=X^{\left(i\right)}*\;\beta +\alpha^{\left(i\right)}+\varepsilon^{\left(i\right)}$$

where y^(*i*)^ is the *i*th measurement, **X**^(*i*)^ is the *i*th row of the design matrix of fixed effect covariates **X**, β is the vector of fitted coefficients (e.g., slopes or contrasts between group means), α^(*i*)^ is the *i*th random intercept (a function of subject identity), and ϵ^(*i*)^ is the *i*th error. Both α and ϵ are assumed to be normally distributed random variables, which have means of 0 and variances described by $\sigma_{\alpha }^{2}$ and $\sigma_{\varepsilon }^{2},$ and these variance terms are fitted as part of the likelihood-based modeling procedure. The intraclass correlation coefficient is defined as:

$$ICC=\frac{\sigma_{\alpha }^{2}}{\sigma_{\alpha }^{2}+\sigma_{\varepsilon }^{2}}$$

and ranges between 0 and 1. Figure 1 illustrates how the ICC measures the degree of consistency between subject measurements. If the response variable *y* is adjusted for its expected value based on fixed effects as y-E(y), where E(y) = X*β (e.g., a group mean), the resulting data will be centered around 0. In the simplest scenario, the random intercept will represent the average of subject values after accounting for E(y). If measurements are consistent, then respective individuals will vary tightly around this intercept (Figure 1A) after adjustment. If this is the case, very little variance between subjects will remain after adjusting for these intercepts, $\sigma_{\alpha }^{2}\gg \sigma_{\varepsilon }^{2}$, and the ICC will approach 1.0. However, if individuals vary inconsistently, then their intercepts after adjusting will be close to 0. In other words, it will be difficult to predict where, with respect to the group estimate, an individual will be encountered from measurement to measurement (Figure 1B) and the fitted intercepts will do little to account for the remaining variance. In this scenario, $\sigma_{\alpha }^{2}\ll \sigma_{\varepsilon }^{2}$ and the ICC will approach 0. Although the fitted values of $\sigma_{\alpha }^{2}$ and $\sigma_{\varepsilon }^{2}$ derived from the mixed model are point estimates, using a bootstrap approach, we are able to assign confidence intervals to these values, and thus the value of the ICC.

**Figure 2:**
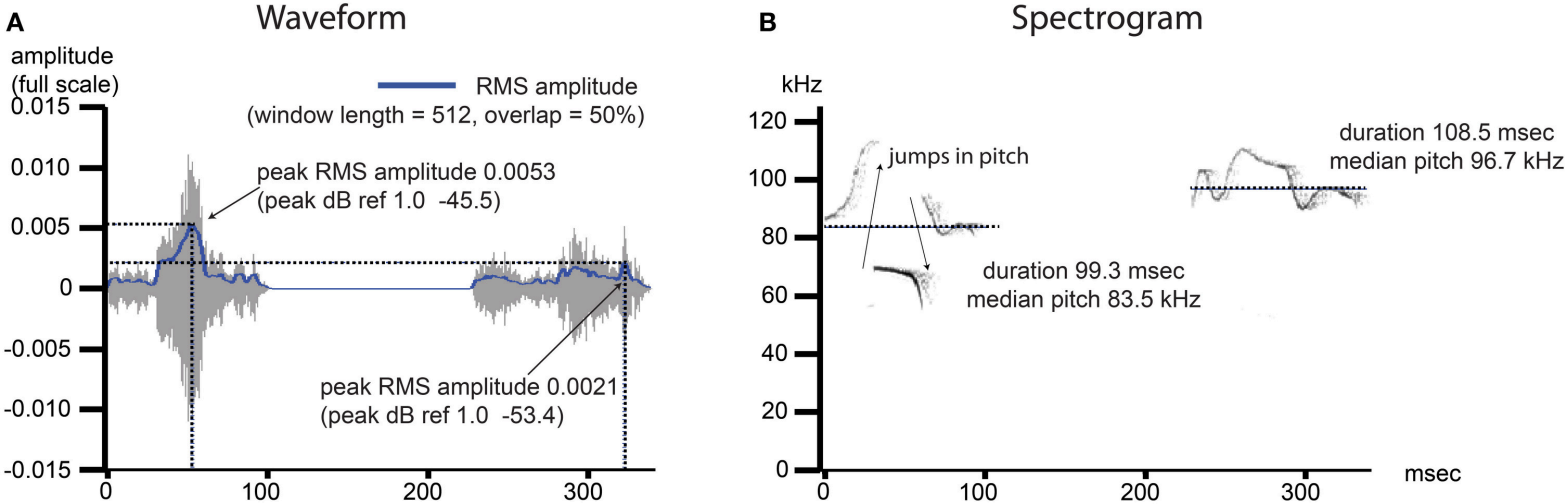
**USV features under investigation include commonly measured features from the time and frequency domain**. Example image shows two pup isolation-induced ultrasonic calls. **(A)** Waveform: the time domain data for the noise- and frequency-filtered calls. A root mean squared (RMS) amplitude envelope is determined for each calls, and the peak power from this envelope (*power* = *amplitude*^2^) is determined and reported as dB ref 1.0. The average of this measurement is determined over all calls, per recording, as well as the variability in this measurement, reported as the standard deviation divided by the mean (coefficient of variation). **(B)** Spectogram: the frequency domain data for the noise- and frequency-filtered calls. The presence of a pitch jump is determined by an instantaneous change in the frequency of maximum power ≥10 kHz, and the fraction of all calls containing at least 1 such jump was computed. The median value of the pitch (kHz) as well as the duration (ms) were determined, and both the average over all calls by recording as well as the coefficients of variation (sd/mean) representing the variability in these measurements over all calls, were computed.

Using the ICC, we sought to explore consistency across some of the most commonly estimated features of USV in the time and frequency domains (Figure 2). In addition to the call production rate, we also looked at the fraction of calls with pitch jumps (≥10 kHz), as well as the duration, median pitch, and peak power. Because animals differ in the number of calls they produce, duration, pitch, and power estimates were computed as either an average over all calls for each recording, or the variability over all calls expressed as the coefficient of variation (standard deviation/mean). These features were selected based upon their salience in previous studies of USV. Pup calls are distinguishable from adult USV (Liu et al., 2003) and pitch and duration of these calls elicit maternal neuronal response and search behavior (Ehret and Haack, 1982; Ehret, 2001; Liu and Schreiner, 2007). Using these call features as our dependent variables, the ICC represents a summary statistic describing how consistently an individual's place in the population varies across measurements from time point to time point. It helps to address, for example, whether an animal producing the longest or loudest calls on day 5 is also producing the longest or loudest calls on day 7, relative to the rest of the population.

Thus, we first analyzed a discovery cohort (PCS) gleaned from 3 datasets, 2 from C57BL/6J animals and 1 from FVB/AntJ animals. In our statistical model, we controlled for effects of: strain (genetic effects and shared environment), age (postnatal day), and relative animal size (weight normalized by strain and postnatal day). We also considered other factors such as sex and litter size, however exploratory preliminary analysis did not determine statistically significant effects for these factors and they were excluded from the model [not shown]. Descriptive statistics for call features between groups and across days in the PCS are shown in Supplemental Table 1.

**Figure 3:**
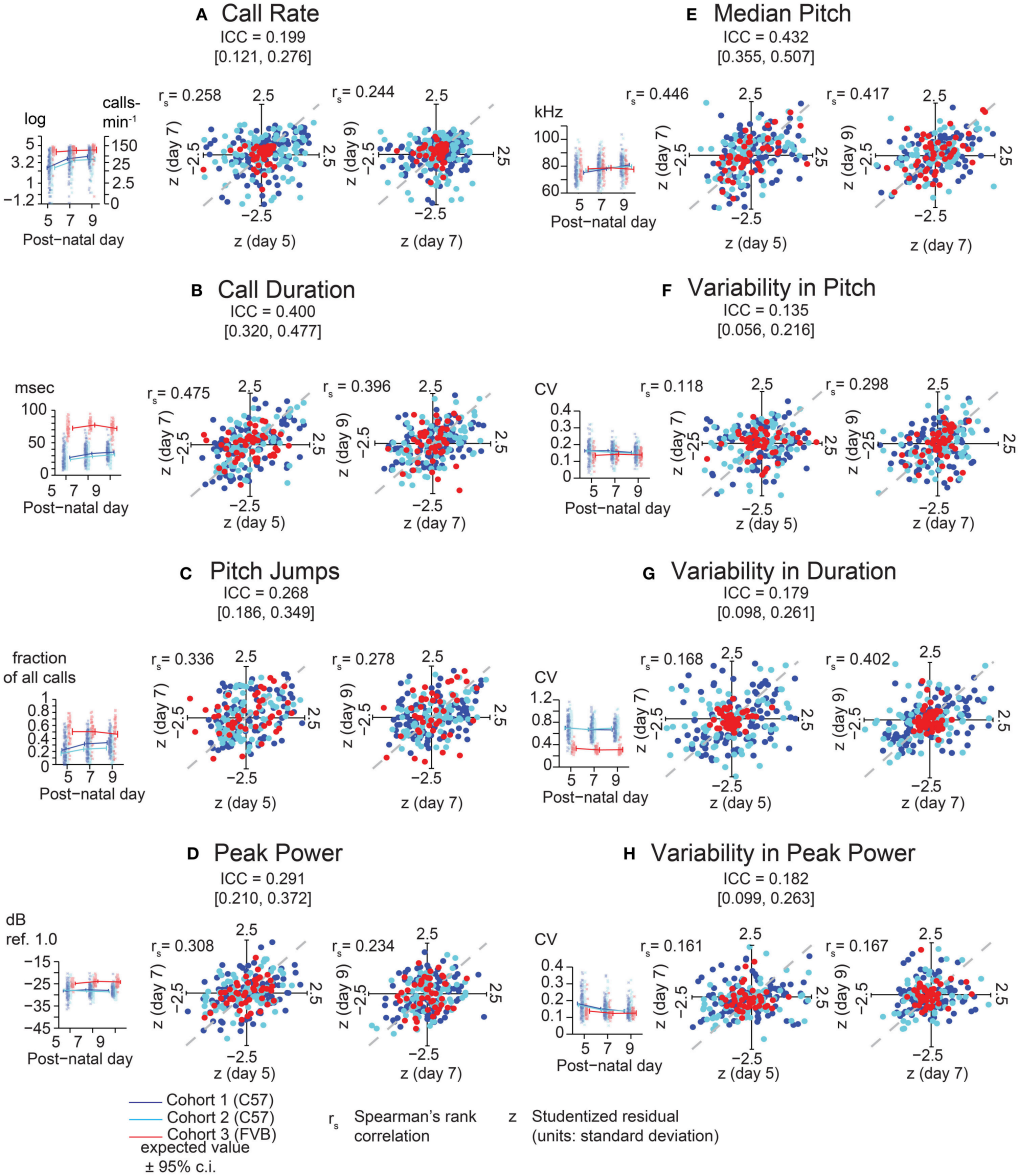
**Lack of Strong consistency across pup USV features in Pooled Cohort Study (PCS)**. Each panel shows: (left) the value of the ICC with its boostrapped 95% confidence interval, the data, with bee plots of individual animals, and trend lines, color-coded by cohort [blue, Cohort 1 (C57BL/6J, *N* = 133); cyan, Cohort 2 (C57BL/6J, *N* = 105); red, Cohort 3 (FVB/AntJ *N* = 47)]; showing expected values from the LMM [fixed effects only, *w* = 0 (average weight)] ± bootstrapped 95% confidence intervals on regression estimates (right) Studentized residuals (z) after adjusting for fixed effects plotting day 5 vs. day 7, and day 7 vs. day 9, and their respective Spearman rank correlation coefficients. The ICC is a summary statistic for each USV feature's consistency, but note that Spearman rank correlation coefficients are typically within or near the range of the respective ICC's confidence bounds. **(A)** Call Rate (calls-min^−1^). LMM was fitted on $\log\left(\frac{counts+1}{minutes}\right)$ (abbreviated “log” on y-axis) with data (left panel) shown alongside linear scale values for ease of interpretation. LMMs can tolerate missing data points, and not all animals have data on all three time points due to pup death. Residual plots and associated correlation coefficients were only computed for animals with data on all three time points: *N* = (1) 119, (2) 101, (3) 47. **(B)** Call Duration (averaged over all calls, milliseconds). **(C)** Pitch Jumps (fraction of all calls). **(D)** Peak Power (averaged over all calls , dB ref. 1.0). **(E)** Median Pitch (averaged over all calls, kHz). **(F)** Variability in Pitch. **(G)** Variability in Duration. **(H)** Variability in Peak Power **(F–H)**: [coefficient of variation (σ/μ) over all calls]. Other than call rate **(A)**, other features of USV **(B–H)** were only computed for animals possessing at least 10 calls [Day 5: *N* = (1) 114, (2) 90, (3) 47 | Day 7: *N* = (1) 122, (2) 99, (3) 47 | Day 9 *N* = (1) 116, (2) 98, (3) 46]. LMMs fitted in R using *lme4* with models in Wilkinson notation as: feature $\sim \underset{\text{fixed effects}}{\underline{cohort*w*(d+d^{2})}}+\underset{\text{random effect}}{\underline{\left(1|id\right)}}$ where cohort is categorical, *w* is a z-score of the animals weight by cohort and day reflecting its relative size, and *d* is postnatal day centered around day 7, fitting both linear and quadratic terms, and (1|*id*) is a random intercept for each animal. Residual plots for **(B–H)** had at least 10 calls and data for all time points, *N* = (1) 98, (2) 80, (3) 46. Highest ICC was observed for call duration with ICC = 0.400 [0.320, 0.477], with rank correlations of 0.475 on day 7 vs. day 5, and 0.396 on day 9 vs. day 7, and median pitch ICC = 0.432 [0.355, 0.507], with correlations of 0.446 on day 7 vs. day 5 and 0.417 on day 5 vs. day 9. Most features of USV have values off ICC near 0.3 or 0.2 indicating overall low levels of day-to-day consistency. Gray dotted lines show correlation of 1.0 for comparison.

Examining the consistency of these eight features, we found that each generally showed low consistency across days for a given animal (Figure 3). Specifically, the most widely assessed variable in studies of pup USV, call rate (Figure 3A), showed an ICC of 0.20 (c.i. [0.12, 0.27]) indicating low consistency over postnatal days, and Studentized residuals *z* show rank correlations of 0.26 (Day 5 vs. Day 7) and 0.24 (Day 7 vs. Day 9) which are within the range of the ICC interval. In contrast, average call duration (Figure 3B) showed a marginally higher ICC at 0.40 (c.i. [0.32, 0.48]) and residuals showed rank correlations of 0.48 and 0.40 for Days 5/7 and Days 7/9 respectively. However, aside from call duration and median pitch (Figure 3E), most features showed low consistencies with ICC values and rank correlations < 0.3.

**Figure 4:**
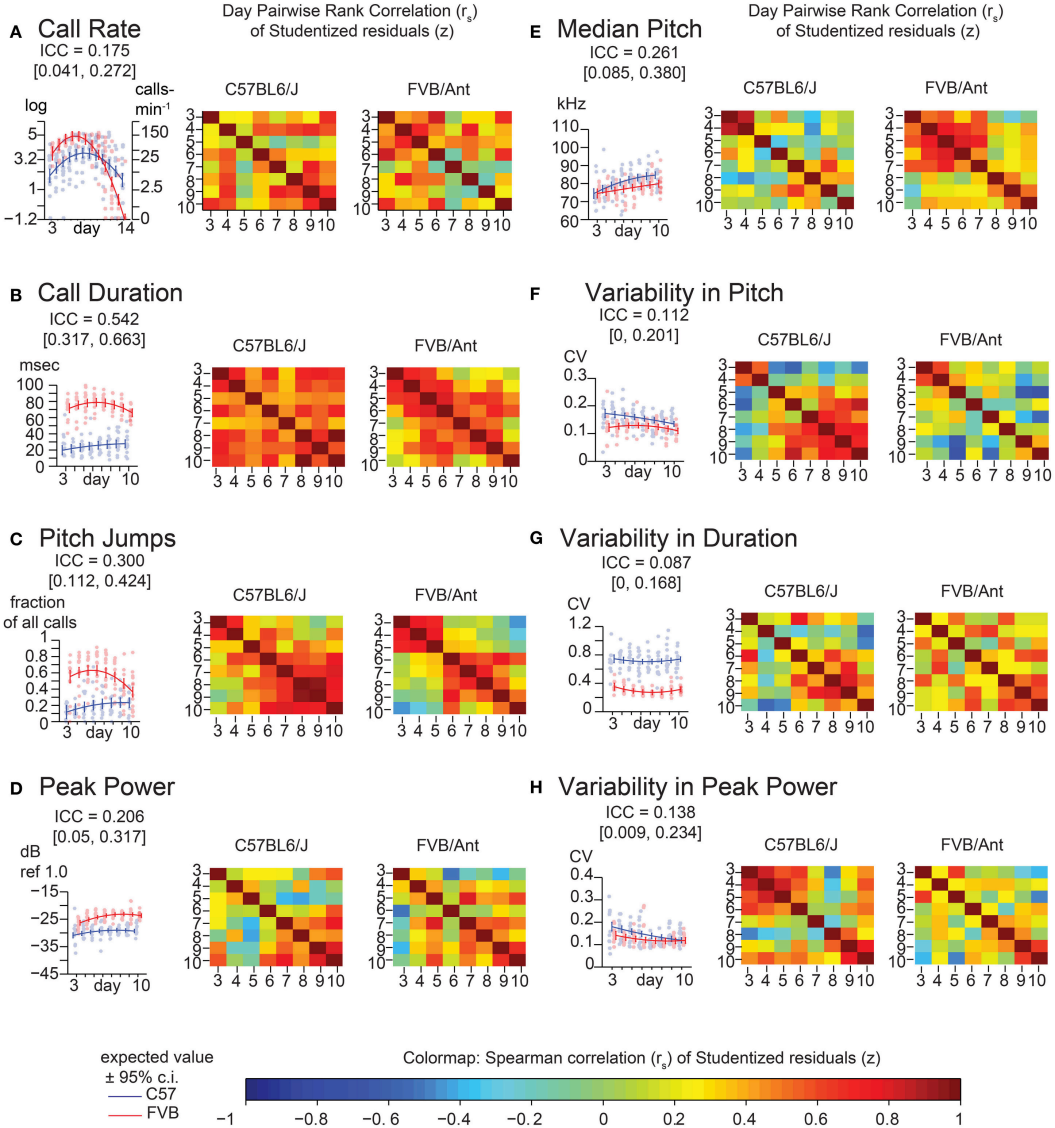
**Lack of strong consistency across pup USV features in Time Course Study (TCS)**. Univariate linear mixed models (LMMs), ICC values, and residuals, and associated correlation coefficients were computed for data from 2 litters each of C57BL/6J (*N* = 8 & 5) and FVB/AntJ (*N* = 8 & 5) as in Figure 2, measured each day postnatally between days 3–14. Each panel shows: (left) value of the ICC, data, with expected values (*w* = 0, average weight) and 95% confidence intervals above beeplots and trendlines as in Figure 2, (right) pairwise day-by-day Spearman correlation of Studentized residuals after adjusting for fixed effects, as in Figure 2, displayed as heat maps (range: blue *r*_*s*_ = −1.0, red *r*_*s*_ = 1.0). **(A)** Call rate. Data were transformed as $\log\left(\frac{counts+1}{minutes}\right)$ (abbreviated “log” on y-axis) as in Figure 3, with linear scale values shown for ease of interpretation. Note that modeling day as both linear and quadratic terms allows for prediction of the characteristic rise and fall in call rate observed through the first 2 weeks of life. Overall ICC is low and within range of PCS (ICC = 0.175 [0.041, 0.272]), however heatmaps reveal a density of stronger correlation near the respective peak for each strain. Beyond day 10, most FVB/AntJ animals did not exhibit >10 calls per sonogram, so graphs in B-H, and all correlation heat maps only show data between days 3–10. **(B)** Call Duration. **(C)** Pitch Jumps. **(D)** Peak Power. **(E)** Median Pitch. **(F)** Variability in Pitch. **(G)** Variability in Duration. **(H)** Variability in Peak Power. Heatmaps showing residual Spearman cross-correlation for **(B–H)** had at least 10 calls and data for all time points, *N* = 9 (C57BL/6J), 13 (FVB/AntJ). As in the PCS (Figure 2), call duration **(B)** shows the highest ICC 0.542 [ 0.317, 0.663 ] with higher levels of correlation day to day across all days. Median pitch did not reproduce the result in Figure 2 when all days were taken into account though slightly overlaps the confidence interval: ICC = 0.261 [0.085, 0.380]. Note both strains show an inflection in their correlations for fraction of calls with pitch jumps near day 5–6 for FVB/AntJ and day 4–5 for C57BL/6J which may indicate that something around this time is important for the development of this kind of call.

To replicate these findings and improve the temporal resolution of these data, we recorded animals from two litters each of C57BL/6J and FVB/AntJ every day between postnatal days 3–14 (TCS). Descriptive statistics for this group of animals is shown in Supplemental Table 2. Since most FVB/AntJ animals did not call beyond postnatal day 10, only 3–10 are considered for USV features other than call rate. These data are shown in Figure 4. Features of USV in the TCS largely recapitulated the overall low consistency exhibited by animals in the PCS. Pairwise, day-by-day, rank correlations of residuals are shown as heatmaps. ICC is an aggregate measure across all time points. Although on the whole call rate shows low consistency, inspecting the heat maps (Figure 4A), one can observe an increase in pairwise correlation near the peak of vocalization behavior [just before postnatal day 5 for FVB/AntJ (Spearman's rank correlation *r*_*s*_ days 4 and 3 = 0.54, days 5 and 4 = 0.63) and just after postnatal day 7 (Spearman's rank correlation *r*_*s*_ days 8 and 7 = 0.58, days 9 and 8 = 0.75) for C57BL/6J]. Thus, call rate appears to show a trend toward increased stability at specific times. Interestingly, the pattern of correlation over time is different across other features of USV. Strong correlation of the median pitch (Figure 4E) for C57BL/6J appears to be restricted to an early time window (days 3–4), which degrades later in development, while FVB/AntJ shows this stronger correlation for a wider time window (days 3–7). Both strains show similar increased consistency in peak power later in development (after postnatal day 7). Thus, features of USV, while on the whole inconsistent across developmental time, show windows of stability which depend on the feature and the strain.

The values of ICC are tabulated for the PCS and TCS in Table 1. The point estimates of ICC for each USV feature between the PCS and TCS are replicable (Pearson's *R* = 0.77, *p* = 0.025, note largely overlapping confidence intervals for most variables), although some features such as median pitch did not replicate well as indicated by poorly overlapping confidence intervals. Considering results from both datasets, after predicting an animal's response using fixed effects, where in the distribution the animal will lie above or below this estimate is not strongly consistent from day to day. However, although overall consistency is low for features of USV, the actual estimates of the ICC values are reproducible across studies, describing a seemingly robust property of these features. This is remarkable, considering that the PCS and TCS differ markedly in terms of their size, composition, and number of time points.

**Table 1:** **Values of the ICC and Confidence Intervals Computed in the PCS and TCS**.

**Feature**	**PCS**			**TCS**		
	**Estimate**	**Lower 95%**°	**Upper 95%**°	**Estimate**	**Lower 95%**°	**Upper 95%**°
Log call rate	0.199	0.121	0.276	0.175	0.041	0.272
Duration	0.400	0.320	0.477	0.542	0.317	0.663
Calls with pitch jumps	0.268	0.186	0.349	0.300	0.112	0.424
Median pitch	0.432	0.355	0.507	0.261	0.085	0.380
Peak power	0.291	0.210	0.372	0.206	0.050	0.317
Variability in duration	0.179	0.099	0.261	0.087	0	0.168
Variability in pitch	0.135	0.056	0.216	0.112	0	0.201
Variability in peak power	0.182	0.099	0.263	0.138	0.009	0.234

*°95% confidence intervals computed from paramateric bootstrap (N = 1 × 10^5^) on linear mixed model parameters (see Section Methods)*.

### 3.2. Consistency of USV features within recording sessions

The relatively low consistency observed in the preceding section over developmental time could arise because USV is highly susceptible to uncontrolled intrinsic or environmental covariates, present at the time of experimentation, which perturb each individual animal's response for the duration of the recording. Alternatively, low consistency could be due to the inherent noisiness of features of USV. If the latter were the case, we hypothesized that, even within a recording session, we would find that USV features were inconsistent across the course of the session. If so, ICC computed across a recording session should be similar to ICC computed across development. If, however, consistency of USV features were higher within a recording session compared to across sessions, then we hypothesize instead that USV itself is not inherently noisy, but rather reflects perturbation of the pup's state at the time of recording by some unmeasured developmental or environmental variable.

**Figure 5:**
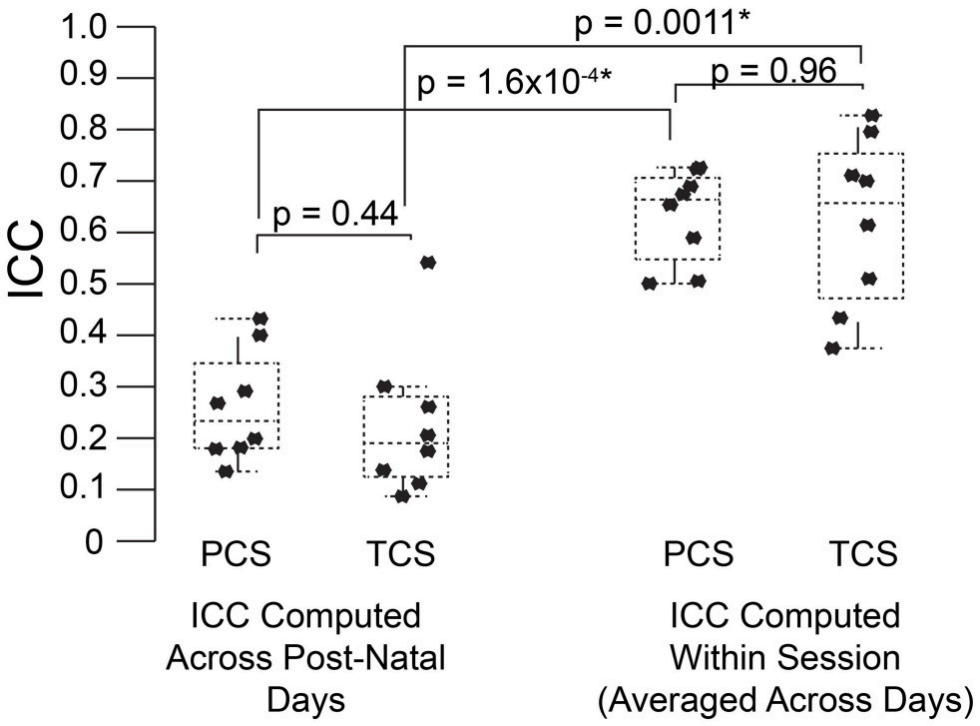
**USV features show higher consistency within within sessions than between sessions**. ICCs were recomputed within session on each postnatal day, using 1 min bins through the recordings (3 for each recording) as the repeated measure instead of postnatal day, in both the PCS and the TCS. Univariate LMMs were fitted using the model *feature*~*bin* *strain *w where w is day/strain z-score of animal's weight as previously. Data are shown for (left) ICC values for all 8 USV features computed across postnatal days (see Figures 2, 3) and (right) computed within session. Overlaid on data points are box plots. Horizontal line represents the median, and box represents lower and upper quartiles (25% (Q1) and 75% (Q3)), with whiskers extending to most extreme datapoints not exceeding 1.5 × the interquartile range. Significant differences in ICC were detected for within session vs. between days (Mann Whitney *p* = 1.6 × 10^−4^, TCS *p* = 0.0011) with the median ICC being 2.8-fold higher within session than across days for the PCS, and 3.3-fold higher in the TCS. Fold increases in ICC in the PCS and TCS $\frac{within\;session}{across\;days}$ were largely reproducible: call rate 3.3-fold (PCS), 3.5-fold (TCS), call duration 1.8-fold (PCS), 1.5-fold (TCS), pitch jumps 2.6-fold (PCS & TCS), median pitch 1.7-fold (PCS), 2.7-fold (TCS), peak power 2.3-fold (PCS), 3.4-fold (TCS) variability in pitch 4.4-fold (PCS), 4.6-fold (TCS), variability in peak power 2.8-fold (PCS), 2.7-fold (TCS), though variability in duration was less reproducible (2.8-fold in the PCS, and five-fold in the TCS). Linear correlation in fold change between PCS and TCS was *R* = 0.68, and 0.83 if variability in duration is omitted. We also did not detect a significant difference in the magnitude of ICC values between PCS and TCS either within session or across days (ICC across postnatal days, PCS vs. TCS, Mann Whitney *p* = 0.4418; ICC within session, averaged across days, PCS vs. TCS Mann Whitney *p* = 0.96). Thus, the ICC and changes to the ICC when computed within recording session vs. across development appear to be robust calculations for these USV features, despite the fact that the PCS and TCS differ widely in the number of individual animals, the number of time points.

To address this question, we computed the ICC in the PCS and TCS on each postnatal day within recordings, where repeated measures consisted of 1 min bins through the 3 min recording. In addition to the fixed effects of strain and size modeled previously, we also controlled for the effect of bin, as the pup's temperature may change through the course of the recording, and temperature has been shown to have an effect on aspects of USV (Okon, 1970; Branchi et al., 2001). The estimates of ICC computed across bins by day in the PCS are shown in Table 2, and for the TCS in Table 3. ICC values are as much as three-fold higher when computed across bins than when computed across developmental time, and these results are summarized in Figure 5 (TCS-Within Session vs. TCS across days Mann Whitney *p* = 0.0011, PCS-Within Session vs. PCS across days *p* = 1.6 × 10^−4^). ICC values computed within bins and averaged across days for each study are tabulated in Table 4. Again, the results are strongly reproducible (Pearson's *R* = 0.95 , *p* = 3.1 × 10^−4^) across studies.

**Table 2:** **Values of the ICC and Confidence Intervals Computed in the PCS across minute bins**.

**Feature**	**Day**	**Estimate**	**Lower 95%°**	**Upper 95%°**
Log call rate	5	0.614	0.551	0.668
	7	0.673	0.617	0.722
	9	0.675	0.617	0.724
Duration	5	0.696	0.632	0.754
	7	0.725	0.671	0.771
	9	0.748	0.697	0.792
Calls with pitch jumps	5	0.729	0.671	0.781
	7	0.652	0.588	0.708
	9	0.687	0.627	0.739
Median pitch	5	0.690	0.625	0.748
	7	0.712	0.656	0.761
	9	0.777	0.731	0.816
	9	0.700	0.642	0.751
Peak power	5	0.619	0.544	0.688
	7	0.703	0.646	0.753
	9	0.700	0.642	0.751
Variability in duration	5	0.559	0.476	0.636
	7	0.501	0.422	0.574
	9	0.441	0.357	0.519
Variability in pitch	5	0.649	0.578	0.714
	7	0.524	0.447	0.594
	9	0.595	0.524	0.659
Variability in peak power	5	0.427	0.331	0.517
	7	0.480	0.399	0.554
	9	0.610	0.541	0.672

*°95% confidence intervals computed from paramateric bootstrap (N = 1 × 10^5^) on linear mixed model parameters (see Section Methods)*.

**Table 3:** **Values of the ICC and Confidence Intervals Computed in the TCS across minute bins**.

**Features**	**Day**	**Estimate**	**Lower 95%°**	**Upper 95%°**
Log call rate	3	0.571	0.299	0.750
	4	0.441	0.150	0.656
	5	0.563	0.291	0.745
	6	0.452	0.163	0.666
	7	0.791	0.605	0.887
	8	0.497	0.213	0.698
	9	0.816	0.648	0.902
	10	0.783	0.594	0.883
Duration	3	0.860	0.695	0.938
	4	0.784	0.570	0.897
	5	0.851	0.686	0.935
	6	0.863	0.725	0.934
	7	0.714	0.482	0.846
	8	0.905	0.806	0.955
	9	0.867	0.737	0.931
	10	0.776	0.574	0.886
Calls with pitch jumps	3	0.793	0.572	0.906
	4	0.782	0.565	0.897
	5	0.779	0.552	0.900
	6	0.773	0.566	0.887
	7	0.795	0.607	0.893
	8	0.821	0.651	0.911
	9	0.790	0.606	0.888
	10	0.832	0.669	0.917
Median pitch	3	0.696	0.406	0.857
	4	0.736	0.490	0.873
	5	0.507	0.152	0.750
	6	0.731	0.499	0.864
	7	0.809	0.629	0.901
	8	0.671	0.412	0.828
	9	0.800	0.622	0.894
	10	0.738	0.513	0.864
Peak power	3	0.862	0.696	0.939
	4	0.738	0.492	0.874
	5	0.630	0.311	0.823
	6	0.439	0.114	0.680
	7	0.721	0.492	0.851
	8	0.804	0.623	0.902
	9	0.817	0.650	0.903
	10	0.588	0.301	0.774
Variability in duration	3	0.585	0.251	0.794
	4	0.000	0.000	0.313
	5	0.650	0.343	0.833
	6	0.651	0.381	0.817
	7	0.267	0.000	0.530
	8	0.887	0.772	0.946
	9	0.161	0.000	0.420
	10	0.271	0.000	0.545
Variability in pitch	3	0.831	0.639	0.924
	4	0.339	0.000	0.623
	5	0.379	0.010	0.667
	6	0.759	0.545	0.880
	7	0.102	0.000	0.375
	8	0.065	0.000	0.355
	9	0.257	0.000	0.509
	10	0.265	0.000	0.537
Variability in peak power	3	0.394	0.023	0.673
	4	0.433	0.086	0.689
	5	0.364	0.000	0.655
	6	0.402	0.077	0.652
	7	0.511	0.216	0.717
	8	0.743	0.521	0.870
	9	0.581	0.307	0.758
	10	0.652	0.387	0.815

*°95% confidence intervals computed from paramateric bootstrap (N = 1 × 10^5^) on linear mixed model parameters (see Section Methods)*.

**Table 4:** **ICC in the PCS and TCS across Minute Bins: Averages and Standard Deviations**.

**Feature**	**PCS**		**TCS**	
	**Average**	**S.D**.	**Average**	**S.D**.
Log call rate	0.654	0.035	0.614	0.158
Duration	0.723	0.026	0.827	0.063
Calls with pitch jumps	0.689	0.039	0.796	0.021
Median pitch	0.726	0.045	0.711	0.094
Peak power	0.674	0.048	0.700	0.141
Variability in duration	0.501	0.059	0.434	0.302
Variability in pitch	0.589	0.063	0.510	0.137
Variability in peak power	0.506	0.094	0.375	0.281

Thus, these data support the hypothesis that most features of USV are not inherently inconsistent, but instead inconsistencies across development may arise from unknown variables affecting the animal's state at the time of recording. Examining results from both PCS and TCS indicate our estimates of ICC both between and within sessions are robust to relatively large differences in experimental design, such as the number of time points considered and sample size.

### 3.3. Consistency of features of USV in adult male–female C57BL/6J dyads

In the preceding sections, we have shown that there is overall low consistency across the features of USV examined in mouse pups across recording sessions, yet that consistency is high within a recording session. We next examined whether adult male USV was also primarily “state” dependent or “trait” dependent. We measured USV from 47 adult male animals on two test days with a different unfamiliar female on each day, made up entirely of C57BL/6J animals. This dataset differs in a few fundamental ways: (1) the stimulus is the presentation of an adult female mouse to the male, rather than isolation of pups from the dam, (2) the recordings are dyadic. Although historically it has been suggested that in such a paradigm only the male is vocalizing (Warburton et al., 1989), recently it has been shown that an appreciable number of vocalizations can be attributed to the female (Neunuebel et al., 2015). We make no strong claims that our data represent something unique to male behavior. Finally, the number of measurements differs importantly in that for pups each time point represents potentially a different developmental stage, while for the adults time points are at the same developmental stage. Linear modeling in either case, however (either modeling post natal day or adult test day as a fixed effect), allows for the effect of postnatal age or test session to be regressed before assessing consistency. Consistency itself (the ICC) is thus still comparable as it resides on the same scale representing the ratio of variance amongst individuals' intercepts to the combined variance of random effects and error. For our adult recordings, as there are only two time points, the ICC values will be expected to be near the simple pairwise correlation across test days. Pups during development are changing in a rapidly dynamic fashion, which we do not discount. However, because the interpretation of the ICC is the same in either case (consistent or inconsistent), we believed the comparison between the datasets serves to identify which features of USV may stabilize later in life, and which may remain dynamic.

**Figure 6:**
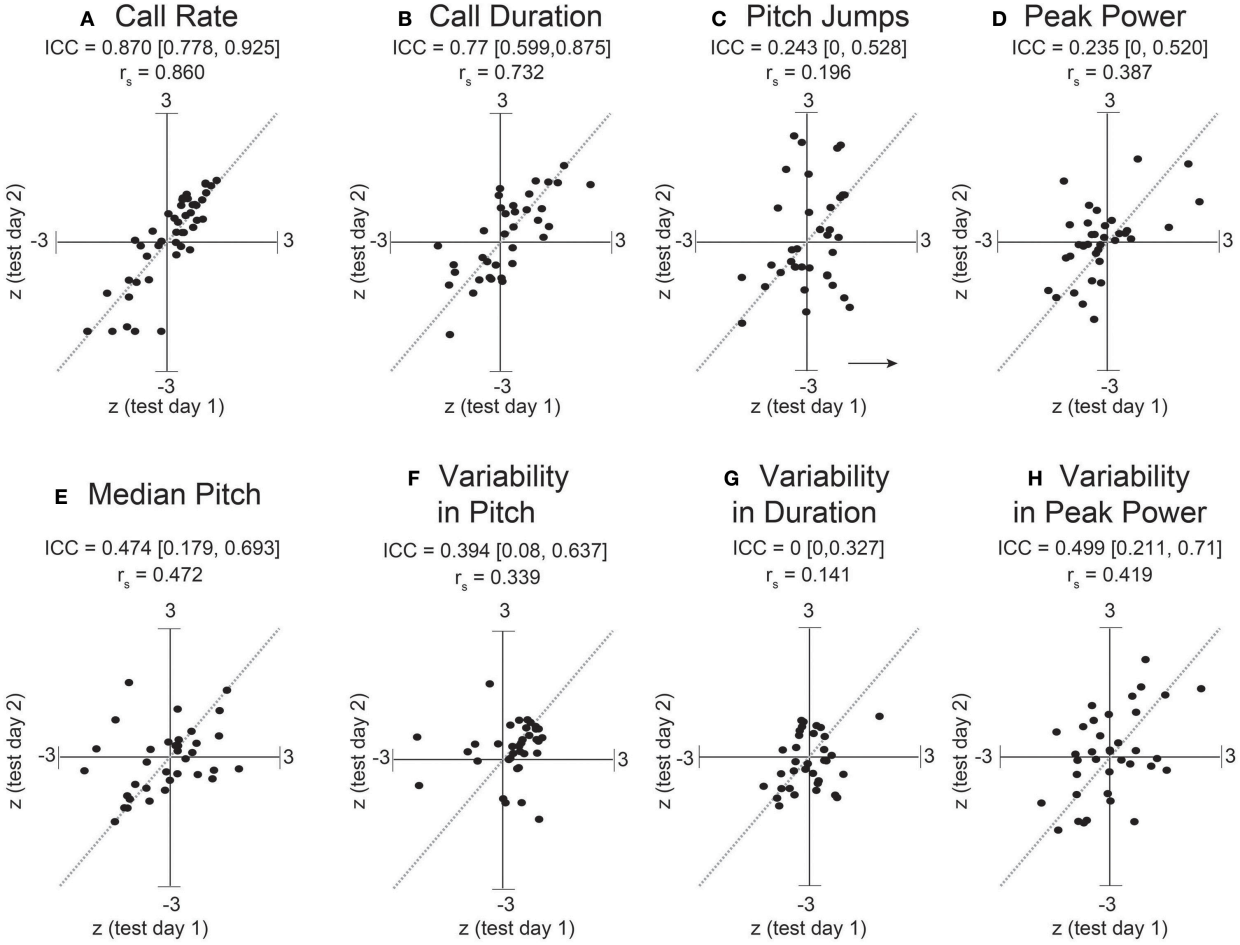
**Stronger consistency in some adult USV features across sessions**. ICC and Spearman correlations computed for adult C57BL/6J M-F dyads across 8 features of USV. ICC and rank correlations were computed for 47 male-female pairs between 7 and 11 weeks of age, in which 47 group-housed males were tested two different days with a unique female each time. LMMs were fitted only using test day as a fixed effect factor: *feature*~*test* *day*+(1|*id*). (Note: with only two time points, we expect the correlation coefficients to be very close to the estimates of the ICC). Studentized residuals (*z*) between test days are shown for **(A)** call rate with LMM fitted for $\log\left(\frac{counts+1}{minutes}\right)$, **(B)** call duration, **(C)** pitch jumps, **(D)** peak power, **(E)** median pitch, **(F)** variability in pitch, **(G)** variability in duration, and **(H)** variability in peak power. Call rate exhibited a much higher consistency (ICC = 0.870, [0.778, 0.925], r_*s*_ = 0.86) than observed for any pairwise day comparison in pup data in Figures 2, 3. Call duration also showed higher consistency (ICC = 0.77, [0.599, 0.875], r_*s*_ = 0.732). However, note other features of USV showed values of ICC and corresponding correlation coefficients which are in the range of those observed for pups across early postnatal development. Thus, most features of USV appear to remain relatively inconsistent from measurement to measurement, although in these data, the adult call rate & call duration appear to be stable features and exhibit trait-like behavior. Gray dotted lines show correlation of 1.0 for comparison.

Dramatically the ICC for adult call rate (Figure 6, Table 5) was much higher than that observed in pups, and even higher than the value obtained within pup sessions (ICC = 0.87, c.i. [0.78, 0.93]), which is also reflected in the rank correlation (*r*_*s*_ = 0.86). Call duration showed values of ICC which were similar to that obtained within session for pups (ICC = 0.77, c.i. [0.60, 0.87], *r*_*s*_ = 0.73). However, with the exception of log call rate, other features of USV, such as the median pitch, peak power, and fraction of calls with pitch jumps, showed ICC values and rank correlations in the range of those obtained for pups. This may indicate that features such as call rate and call duration approach trait-like stability in adult animals, however other features of USV still depend on the state of the animal and its environment. Descriptive statistics for our adult data are presented in Supplemental Table 3.

**Table 5:** **Spearman's Correlation and ICC computed for adult C57BL/6J data**.

**Feature**	**r_*s*_**	**ICC**	**Lower 95%°**	**Upper 95%°**
Log call rate	0.86	0.87	0.78	0.93
Duration	0.73	0.77	0.60	0.87
Calls with pitch jumps	0.20	0.24	0	0.53
Median pitch	0.39	0.24	0	0.52
Peak power	0.47	0.47	0.18	0.69
Variability in duration	0.34	0.39	0.08	0.64
Variability in pitch	0.14	0	0	0.33
Variability in peak power	0.42	0.50	0.21	0.71

*°95% confidence intervals computed from paramateric bootstrap (N = 1 × 10^5^) on linear mixed model parameters (see Section Methods)*.

## 4. Discussion

In this investigation, we have examined datasets generated in our laboratory in order to understand the extent to which features of vocalization show consistent inter- and intra-individual patterns across measurements. In young pups, we have found, in general, that across development most features of USV such as the call rate do not show consistent patterns across an individual's measurements, though some such as call duration show a larger degree of consistency. The estimates of consistency in our pup data were largely reproduced when examined in a replication cohort which increased the number of time points across development sampled. When looking within a session, pups across development show a much higher degree of consistency for most features examined. Thus, we hypothesize that the expression of pup USV, although clearly under the influence of population effects such as strain or age, is highly state dependent. Therefore, we conclude that while the population average may rise or fall due to strain or age, the relative ranks of the pups in the distribution must be influenced by other unmeasured aspects of the animal's state. It could be that some of this influence derives from the litter to which the animal pertains, however we have also estimated ICC at the level of litter and have not found any increased explanation of remaining variance upon inclusion of this hierarchy (not shown). As phenotypic expression in an individual's behavior is a complex integration of its state, and genetic and environmental factors (Hofmann, 2003), a lack of consistency is not entirely surprising. Wild species often display behavioral plasticity in the form of inconsistent individual behavior over time, yet show consistent trends at the group level (Lee and Bereijikian, 2007). The study of trait consistency over time amongst individuals has also been appreciated in the domain of human psychology (Roberts and DelVecchio, 2000) and ecology (Bell et al., 2009), but rarely in laboratory animals. In our mice, however, we did observe that there was an increase in intra-animal consistency near each strain's respective peak of vocalization behavior at least with respect to rate of calling. These time points may represent preferable windows to look for effects due to experimental manipulation as individual animals are performing more predictably from measurement to measurement. By contrast, in our adult dataset, consistency in call rate was dramatically higher than for pups, while pitch related features continued to show low consistency. While adults and pups are in different stages of life and react to their environment differently, there appears to be a similarity that pitch features of USV continue to show dynamic modulation even where other features such as call rate show increased consistency. However, as described in Section Methods and Supplemental Figure 3, our adult data were pooled from a study examining changes to USV in adults after global knockout of the Celf6 gene, in which we did not detect significant genotype effects. Future cohorts of animals, with an increased number of test days, should be examined to discern the reproducibility of any trait stability in call rate or other features. The level of intra-individual variability and overall reaction to changes in the external environment has been shown in adult mice to be explainable to some degree by their level of subordinance/dominance and aggressiveness (Benus et al., 1987) and more recently, rate of calling in adult males has been directly correlated to measures of dominance and social hierarchy in tasks such as the tube test, and manipulation of the prefrontal cortex is able to alter the hierarchical rank order among the mice and concomittantly their rates of ultrasonic calling (Wang et al., 2011). In our study, males were socially isolated from their cage hierarchies for 24 h before test day #1 and up to a week before test day #2, though this may not be sufficient time to perturb the established dominance rank order in these males. For features showing poorer consistency (pitch related features) between test days, our results may be somewhat confounded by not fully knowing the animal originating the calls (male or female), and the fact that the female's estrous state was uncontrolled. It has been claimed that males can pitch modulate their song due to the presence of an alleged competitor male (Arriaga et al., 2012). It is attractive to hypothesize that perhaps the state of the female or her contribution to the dyadic song somehow influences the pitch characteristics, and may explain why there are poorer correlations for these features in our study. It will be interesting to observe what other genetic or pharmacological manipulations are able to change the USV trait consistency of adult mice, which will reveal the potential neurological correlates of how these features are encoded. This very fundamental difference in the source of variability between pup USV and adult USV may explain why so few disease models show a consistent carry-over from pup to adult USV changes. Reviewing just the literature on call rate in autism models in particular, 35 of 41 studies have shown alterations in pups behavior which typically manifests as a decrease in call rate. However, of the models where adult behavior was assayed, only 2 showed carry-over of pup USV phenotype into some kind of adult USV phenotype (Michetti, 2012; Roullet et al., 2013). Thus, whatever the mechanisms are that mediate the alterations in pup USV, these largely do not carry over to call rate in male-female song.

In the current study, we have not subcategorized calls into call types based upon spectral and temporal properties. We have avoided this approach as there is no standard method for call classification. Some methods, such as a method employed to study mice with a humanized Foxp2 gene (Enard et al., 2009), classify by length of call and presence of instantaneous jumps in pitch, while others use jumps exclusively based upon their number and direction(Holy and Guo, 2005; Arriaga et al., 2012). Another commonly employed method involves manual sorting of calls into categories based upon spectral shape (Scattoni et al., 2008a), which integrates information about pitch, the presence of jumps, harmonics, duration, and slope. Yet another method uses an unbiased classification scheme (Burkett et al., 2015). It is not clear the extent to which these different classification schemes represent biologically relevant categories. It has been well-documented that the frequency and frequency modulation of the pitch in rat USV is associated with positive and negative emotionality (Knutson et al., 1998, 2002) and rats will even self-administer or exhibit avoidance of the respective category of calls (Burgdorf et al., 2008). While mice emit USV during ostensibly rewarding circumstances such as mating or juvenile play, it is not clear that individual categories of calls based on any available scheme are associated with either reward or aversion, although it has been shown that mice can distinguish between calls of different categories (Neilans et al., 2014). However, all categorization schemes, either explicitly or implicitly, incorporate some aspect of the presence of pitch jumps in classification, and we have examined this feature, which has been shown to exhibit salience in listening animals (Liu and Schreiner, 2007; Portfors et al., 2009). In neither our pup nor adult datasets did we see high degrees of consistency in the fraction of calls containing pitch jumps. However, it will be interesting to see whether a pup or an adult's repertoire, as categorized by one of the above schemes or some other, has the properties of a stable trait across individuals, or whether it too is highly affected by an animal's state. Some categorization schemes may turn out to be more consistent over multiple measurements than others, and this may be a useful criterion to determine which classification scheme may be measuring a stable biological feature. To enable these and other analyses that would benefit from the availability of a standardized dataset for algorithm testing and optimization, we have provided all of our recordings via the mouseTube online database (https://mousetube.pasteur.fr, under user Michael Rieger). We include raw audio files through this platform along with associated metadata, so that researchers may use this resource to address questions such as the stability of categorical assemblies of call types. Future work remains to assess the relative utility of different categorization schemes and their biological relevance.

## 5. Conclusion

In summary, we present an examination of the consistency of patterns of USV expression among developing and adult mice. We provide reliable estimates for strain, age, and size effects as well consistency across measurements across two strains common used for generating disease models. The state dependence of USV in young mouse pups deserves some attention as there are likely to be neurological and physiological mediators of these states which have not yet been explored. Future research using biometric devices may be able to address the physical condition of the pups at the time of vocalization and how such a condition affects features of the pups behavior. Understanding the variability and consistency patterns of vocalization, we hope, helps future scientists to better plan experiments aimed at evaluating phenotypic changes in disease models, as well as discerning which factors mediate state vs. trait patterns of behavioral expression.

## Author contributions

MR designed and performed research, acquired data, processed and analyzed data, and wrote manuscript. JD and MR revised manuscript.

### Conflict of interest statement

The authors declare that the research was conducted in the absence of any commercial or financial relationships that could be construed as a potential conflict of interest. The reviewer RF and handling Editor declared their shared affiliation, and the handling Editor states that the process nevertheless met the standards of a fair and objective review.

## Supplementary material

Supplemental figures 1, 2 and 3 and the legends can be found in Supplementary Data Sheet 1.
