# Supplementary material for: Analysis of within Subjects Variability in Mouse Ultrasonic Vocalization: Pups Exhibit Inconsistent, State-Like Patterns of Call Production
Source: Front Behav Neurosci. 2016 Sep 28;10:182. doi: 10.3389/fnbeh.2016.00182 (PMC5039195; doi:10.3389/fnbeh.2016.00182)

Supplementary Material: Analysis of within subjects variability in mouse ultrasonic vocalization: pups exhibit inconsistent, state-like patterns of call production

Michael A. Rieger and Joseph D. Dougherty *

*Correspondence:
Joseph D. Dougherty, Ph.D.
jdougherty@genetics.wustl.edu

SUPPLEMENTARY FIGURE LEGENDS

Supplemental Figure 1. Conditional knockout of Celf6 in dopaminergic neurons does not alter features of USV in Cohort 1 of PCS. A mouse model was generated possessing a conditional allele of the Celf6 gene, in which the 4th exon was flanked by loxp sites (Celf6^{flox}, abbreviated "f") in order to allow for Cre recombinase mediated excision using the DAT-Cre mouse, which expresses Cre in dopaminergic neurons under control of the dopamine transporter gene Slc6a3. A cross between conditional Celf6$^{flox/+}$ ("f/+") heterozygotes positive for DAT-Cre and X Celf6$^{flox/flox}$ ("f/f") yielded a cohort of 133 animals constituting Cohort 1 of the PCS: 36 f/+, 23 f/+ Cre+, 42 f/f, 32 f/f Cre+, distributed across 18 litters of median size 8 animals, ranging from 4 to 11 animals per litter. Data are shown by genotype and postnatal day as bee plots for individual animals and boxplots showing the median (circle) and 25% (Q3) -75% (Q1) quartiles, with whiskers extending to most extreme datapoints not exceeding 1.5 x the interquartile range, for: (A) Call rate, (B) Call Duration, (C) Pitch Jumps, (D) Peak Power, (E) Median Pitch, (F) Variability in Pitch, (G) Variability in Duration, (H) Variability in Peak Power. Linear mixed models and likelihood ratio tests were used to detect main effects of genotype, postnatal day, or two-way interaction of genotype and postnatal day. Significant main effects of genotype were not detected for any variable, nor were any significant interactions detected. Collapsing across genotype, significant main effect of postnatal day was detected for: (A) Call rate (fitted as log((calls+1)/minutes), p = 6.5x10^{-19}), which was driven by a significant increase between days 5 and 7 (log fold change: 0.89, 95% c.i. [0.64, 1.13]) as well as a lesser but significant increase between days 7 and 9 (log fold change day 9 - 7: 0.31, 95% c.i. [0.05, 0.56]), (B) Call Duration (msec, average over all calls, p = 6.5x10^{-19}), driven by a significant increase between days 7 and 5 (5.6 msec, 95% c.i [3.4, 7.9]) and a slightly smaller increase between days 7 and 9 (2.6 msec, 95% c.i. [0.4, 4.9]), (C) Pitch Jumps (fraction of all calls, p = 1.2x10^{-9}), driven by a significant increase between days 7 and 5 (0.09 (9% increase), 95% c.i. [0.05, 0.12]) but no significant increase detected between days 7 and 9, (E) Median Pitch (kHz, p = 1.9x10^{-13}), driven by a small but significant increase between days 7 and 5 (3.1 kHz, 95% c.i. [1.78, 4.42]) and a similar increase between days 9 and 7 (2.6 kHz, 95% c.i. [1.25, 3.90]), and (H) Variability in Peak Power (coefficient of variation (standard deviation over all calls/mean, per animal), p = 5.8x10^{-12}), exhibiting a decrease in variability between days 5 and 7 (-0.032, 95% c.i. [-0.043, -0.020]), and smaller amount of decrease between days 7 and 9 (-0.012, 95% c.i. [-0.02, -4.8x10^{-4}]).

Supplemental Figure 2. Conditional knockout of Celf6 in GABA-ergic neurons does not alter features of USV in Cohort 2 of PCS. As in Supplemental Figure 2, conditional knockout of Celf6 in GABA-ergic neurons was performed by crossing Celf6$^{flox/+}$ ("f/+") heterozygotes positive for VGAT-Cre and X Celf6$^{flox/flox}$, where the VGAT-Cre mouse expresses Cre recombinase under the control of the GABA transporter gene Slc32a1. This cross yielded a cohort of 105 animals constituting Cohort 2 of the PCS: 34 f/+, 24 f/+ Cre+, 29 f/f, 18 f/f Cre+, distributed across 15 litters of median size 8 animals, ranging from 2 to 9 animals per litter, with 12 of 15 litters possessing 7 to 9 animals. Data are shown by genotype and postnatal day as bee plots for individual animals and boxplots showing the median (circle) and 25% (Q3) -75% (Q1) quartiles, with whiskers extending to most extreme datapoints not exceeding 1.5 x the interquartile range, for: (A) Call rate, (B) Call Duration, (C) Pitch Jumps, (D) Peak Power, (E) Median Pitch, (F) Variability in Pitch, (G) Variability in Duration, (H) Variability in Peak Power. Linear mixed models and likelihood ratio tests were used to detect main effects of genotype, postnatal day, or two-way interaction of genotype and postnatal day. Significant main effects of genotype were not detected for any variable, nor were any significant interactions detected. Collapsing across genotype, significant main effect of postnatal day was detected for: (A) Call rate (p = 5.54x10^{-9}), showing a significant increase between days 7 and 5 (log fold change in calls/min, 0.75, 95& c.i. [0.47, 1.05]), (B) Call duration (p = 3.03x10^{-7}), showing a significant increase between days 7 and 5 comparable to Supplemental Figure 2 (4.7 msec, 95% c.i. [2.1,7.3], and a small increase between days 9 and 7 (2.5 msec, 95% c.i. [0.01, 5.03]), (C) Pitch Jumps (p = 2.67x10^{-5}), as in Supplemental Figure 2 showing an increase between days 7 -5 in fraction of all calls (0.065, 95% c.i. [0.03, 0.10]) and no significant increase between days 9 and 7, (D) Peak Power (p = 0.005) showing a small decrease between days 7 and 5 (-0.55 dB, 95% c.i. [-1.08, -0.01]) (E) Median Pitch (p = 0.002), exhibiting a small increase between days 7 and 5 similar to Supplemental Figure 2 (2.0 kHz, 95% c.i. [0.42, 3.61] but no significant change between days 9 and 7, (F) Variability in Pitch (p = 0.008), showing a slight decrease in variability between days 7 and 5 (-0.013, 95% c.i [-0.023, -1.9x10^{-3}]) but no change between 9 and 7, and (H) Variability in Peak Power (p = 4.25x10^{-7}), showing a comparable decrease in peak power variability between days 7 and 5 to Supplemental Figure 2 (-0.030, 95% c.i. [-0.043, -0.016]) but no change between days 7 and 9.

Supplemental Figure 3. Global knockout of Celf6 does not perturb USV features in adult male-female dyads. 24 Celf6 WT and 23 KO adult males (7-11 weeks) were tested on two testdays with a different, stranger female each day as described in Methods. Data are shown by genotype and test day as bee plots for individual animals and boxplots showing the median (circle) and 25% (Q3) -75% (Q1) quartiles, with whiskers extending to most extreme datapoints not exceeding 1.5 x the interquartile range, for: (A) Call rate, (B) Call Duration, (C) Pitch Jumps, (D) Peak Power, (E) Median Pitch, (F) Variability in Pitch, (G) Variability in Duration, (H) Variability in Peak Power. Linear mixed models and likelihood ratio tests were used to detect main effects of genotype, postnatal day, or two-way interaction of genotype and postnatal day. No significant effects of genotype nor interactions between genotype and test day were detected. Collapsing across genotype, a significant effect of test day was detected in the case of (B) Call Duration (msec, p = 0.004), in which there was an increase in call duration by 2.1 msec (95% c.i. [0.76, 3.54]) on test day 2. A non-significant trend towards in a increase in (C) Pitch Jumps (fraction of all calls, p = 0.07) was also observed between test days. USV features do not distinguish between vocalizations made by either animal in the dyadic assay and represent an aggregate of both animals, which may effect the precision of some measurements (especially if animals vocalize at the same time).

SUPPLEMENTARY FIGURES

Supplemental Figure 1. Conditional knockout of Celf6 in dopaminergic neurons does not significantly alter features of USV in Cohort 1 of PCS

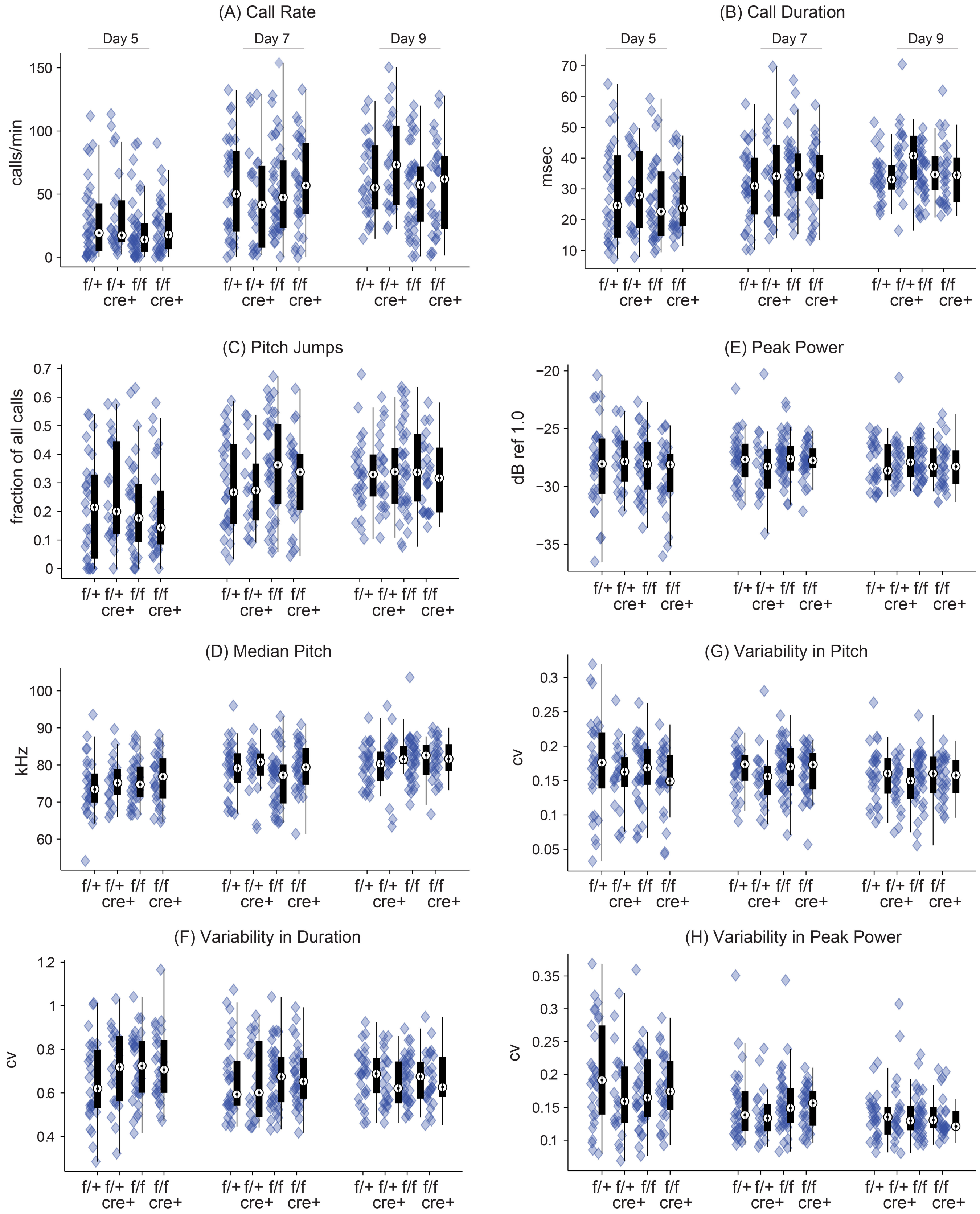

Supplemental Figure 2. Conditional knockout of Celf6 in GABA-ergic neurons does not significantly alter features of USV in Cohort 2 of PCS

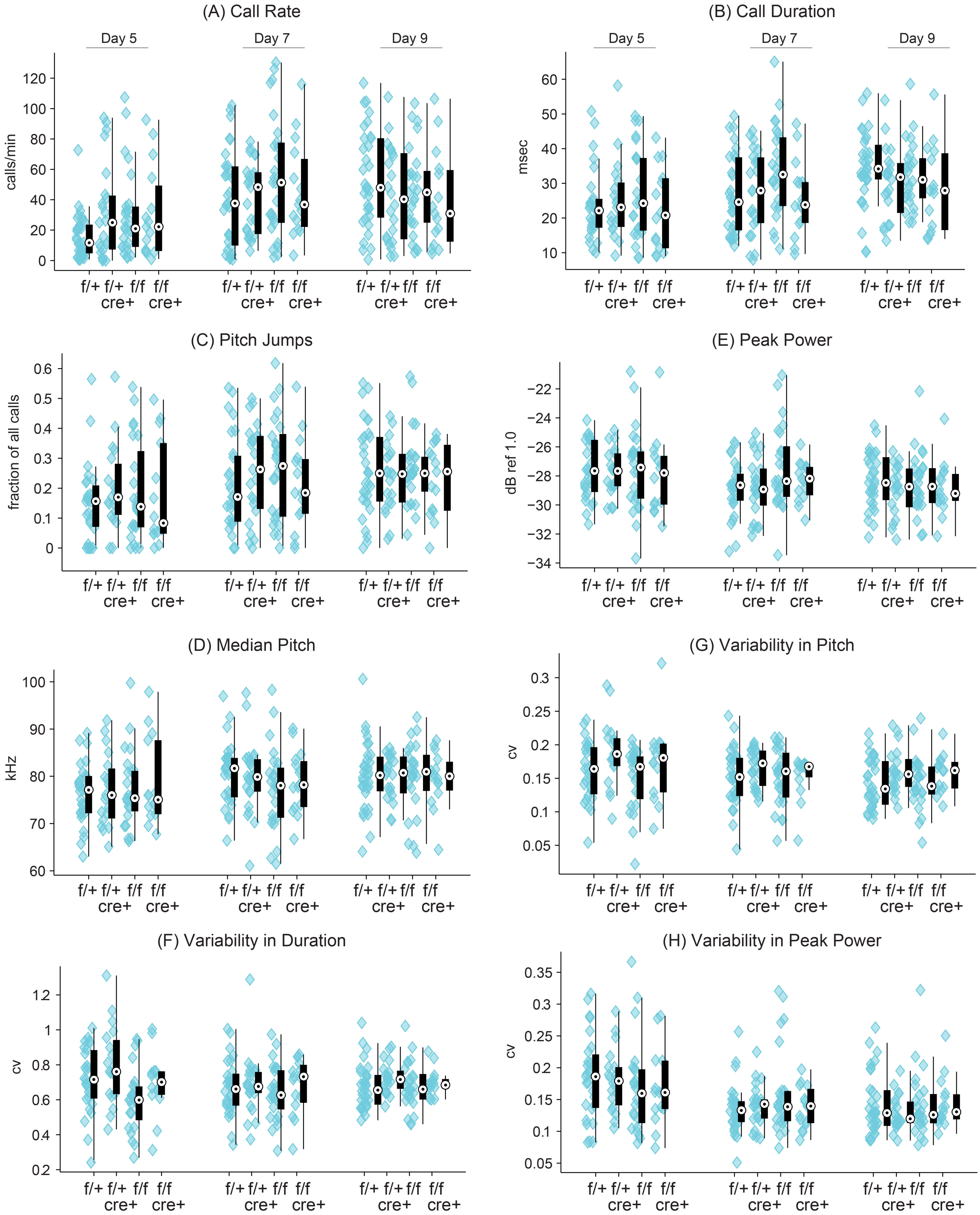

Supplemental Figure 3. Global knockout of Celf6 does not perturb USV features in adult male-female dyads.

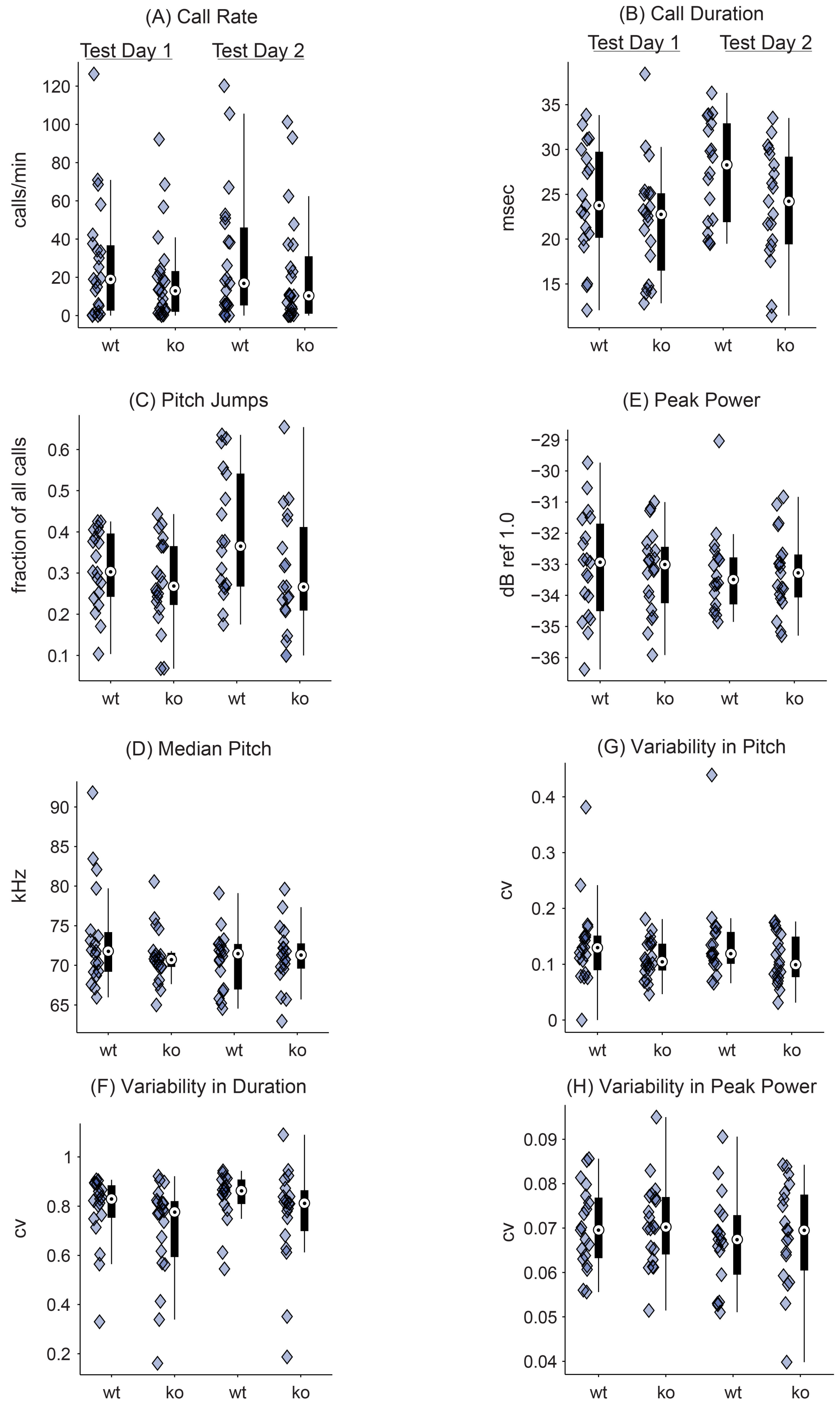

Supplement: Supplementary file 4 [file DataSheet1.pdf]
